# Prediction of successful revision surgery for mesh-related complaints after inguinal hernia and pelvic organ prolapse repair

**DOI:** 10.1007/s10029-023-02748-5

**Published:** 2023-02-08

**Authors:** K. L. C. Van Rest, M. J. C. A. M. Gielen, L. M. Warmerdam, C. R. Kowalik, J. P. W. R. Roovers, W. A. R. Zwaans

**Affiliations:** 1 Department of Obstetrics and Gynecology, Amsterdam University Medical Centers, Location AMC, Meibergdreef 9, 1105AZ Amsterdam, The Netherlands; 2 Amsterdam Reproduction and Development Research Institute, Amsterdam, The Netherlands; 3 Department of Surgery, Máxima Medical Center, Veldhoven/Eindhoven, The Netherlands; 4 Bergman Clinics Vrouw, Amsterdam, The Netherlands; 5 Research Consortium Mesh, Utrecht, The Netherlands; 6 SolviMáx, Center of Excellence for Abdominal Wall and Groin Pain, Eindhoven, The Netherlands; 7 NUTRIM School of Nutrition and Translational Research in Metabolism, Maastricht UMC+, Maastricht, The Netherlands

**Keywords:** Polypropylene, Foreign bodies / complications, Foreign bodies / surgery inguinal hernia, Pelvic organ prolapse, Pain, Postoperative, Surgical mesh

## Abstract

**Purpose:**

With this retrospective case series, we aim to identify predictors for reduction of pain after mesh revision surgery in patients operated for inguinal hernia or pelvic organ prolapse with a polypropylene implant. Identifying these predictors may aid surgeons to counsel patients and select appropriate candidates for mesh revision surgery.

**Methods:**

Clinical records before and after mesh revision surgery from 221 patients with chronic postoperative inguinal pain (CPIP) and 59 patients with pain after pelvic organ prolapse (POP) surgery were collected at two experienced tertiary referral centers. Primary outcome was patient reported improvement of pain after revision surgery. A multivariable logistic regression model was used to specify predictors for pain reduction.

**Results:**

The multivariable logistic regression was performed for each patient group separately. Patients with CPIP had higher chances of improvement of pain when time between mesh placement and mesh revision surgery was longer, with an OR of 1.19 per year. A turning point in chances of risks and benefits was demonstrated at 70 months, with improved outcomes for patients with revision surgery ≥ 70 months (OR 2.86). For POP patients, no statistically significant predictors for reduction of pain after (partial) removal surgery could be identified.

**Conclusion:**

A longer duration of at least 70 months between implantation of inguinal mesh and revision surgery seems to give a higher chance on improvement of pain. Caregivers should not avoid surgery based on a longer duration of symptoms when an association between symptoms and the location of the mesh is found.

## Introduction

The development and introduction of mesh implants in surgery was intended to decrease the recurrence risk of inguinal hernia- and pelvic organ prolapse (POP)- surgery [1–3]. Polypropylene (PP) is a widely used synthetic implant material that is effective and affordable but may also lead to specific complications like (chronic) pain, erosion and exposure. PP is never fully resorbed by the body, and, despite adequate mesh placement, mesh-related pain, excessive mesh shrinkage, mesh exposure and chronic inflammation surrounding the mesh may occur [4–7].

If complications related to the presence of mesh emerge and conservative treatments are insufficiently resolving symptoms, mesh revision surgery may be considered. Such revision involves partial or complete removal of the mesh with the intent to diminish local negative effects of a foreign object and associated tissue reactions, but can result in damage to surrounding visceral organs or recurrence of inguinal hernia or POP. In addition, it has been shown that about 1 out of 3 patients undergoing revision surgery will be indicated for another surgery and therefore will be exposed to complementary risks such as infection and iatrogenic damage. For POP surgery there is no evidence that complete removal of the mesh results in better clinical outcome than partial removal [8–15].

Lack of strict selection criteria for patients and indications for mesh revision surgery likely contributed to heterogeneous results and unclear risks of reoperation. Preoperative indicators that illustrate odds of successful revision surgery are therefore relevant. Earlier research in both medical fields has signaled several predictors and patient characteristics that can aid in predicting whether reoperation will lead to beneficial results. However, since the list is neither exhaustive nor specified for mesh removal, more research is necessary to draw conclusions [16–18].

With a lifelong cumulative incidence of inguinal hernia in adults of 27–42.5% for men and 3–5.8% for women [19] and PP widely used as a primary choice for repair, postoperative pain as a result of the operation in presence of a PP mesh is not rare (approximately 10–12%, decreasing over time, with debilitating chronic pain ranging from 0.5–6%)[19, 20]. Since the 1990s PP is also used in sling surgery for treatment of urinary stress-incontinence [21] and thereafter has been used in various approaches of POP repair. Although surgical techniques have been altered and improved since then, complications as postoperative pain and exposure still have considerable prevalence of 12% and 5–20% respectively [22].

Various countries all over the world have stopped the use of vaginal PP for POP due to complications in long term follow-up. This results in fewer opportunities for large prospective cohort studies in POP repair. Nonetheless, some patients have extensive mesh-related complaints with the need for specialized care.

This retrospective study aims to determine and specify factors that predict outcome of mesh revision surgery in inguinal hernia as well as POP surgery. It hypothesizes that in spite of different mechanical and pathophysiological properties of both treatments, factors can be determined that have sufficient impact on positive outcome of revision surgery and common patterns need to be found. These factors and patterns may aid surgeons in selecting appropriate candidates for mesh revision surgery. We aim at development and implementation of a transparent tool for counseling to further help improve expectation management of surgeon and patient.

## Methods

### Study design

This retrospective study was performed in two medical centers in the Netherlands (SolviMáx, Center of Excellence for Abdominal Wall and Groin Pain, Máxima Medical Center and department of Gynecology, Amsterdam University Medical Center). These tertiary referral centers have extensive experience in (partial) removal of PP implants and have access to a large collection of own recorded patient data. Data were collected from patients with a history of chronic pain following inguinal hernia repair (chronic postoperative inguinal pain; CPIP) or chronic pain or PP exposure following POP surgery with mesh, who underwent subsequent mesh revision surgery. This non-experimental study reviewed the collected data including follow-up, with records between March 2009 and November 2021.

### Ethical permission

All patients considering mesh removal at our outpatient clinic for pain treatment after inguinal hernia or POP repair were asked for signed approval to record data with the aim of future research. Only after consent was given, patient reported measurements were obtained and patient-specific data were retrieved from the electronic patient file for this study.

### Eligibility criteria

To include a vast number of patient records, all patients aged ≥ 18 year with mesh revision surgery for CPIP or complaints after POP repair with a PP mesh, were deemed eligible. The initial mesh placement for hernia repair was performed by the Lichtenstein repair or by pre-peritoneal techniques (i.e., TEP or TAPP) and numerous techniques for pelvic reconstructive surgery (e.g., single incision vaginal mesh, sacral colpopexy, mid-urethral slings and various types of vaginal mesh implants).

*Revision surgery* included all surgeries for complete and partial removal of the implant, transabdominal and transvaginal, open and laparoscopic techniques. For inguinal mesh revision, techniques included meshectomy with or without neurectomy. For POP mesh revision, techniques included the removal of a locking eyelet or anchor, exposure correction and mesh resection by dissection or cleaving. Inclusion criteria were not strict because of the broad variety of techniques used by physicians at placement *and* removal of the mesh.

Exclusion criteria were mesh removal due to an ongoing mesh infection, patients with a malignancy and cognitive impaired individuals. Patients who underwent inguinal remedial surgery more than once for the same side were also excluded, to avoid multiple inclusions of the same patient and therefore skew outcomes or mask potential determinants.

### Data retrieval

Demographics, clinical data and operative notes of the included patients were anonymized and entered into the database. Variables attempted to be extracted from the electronic patient files were age at time of revision surgery, gender, BMI, smoking, pain scores (using Numerical Rating Score (NRS) or Visual Analogue Scale (VAS)), postmenopausal state, sexual activity, vaginal deliveries in the past, number of abdominal surgeries in the past, amount of time between mesh placement and removal, a foreign body feeling, presence of neuropathic pain, comorbidities (including pain syndromes and/or systematic auto-immune disease), duration of presence of pain, use of NSAIDs and/or opioids, type of mesh implant, exposure of mesh, percentage of the mesh removed, number of revision surgeries, intraoperative macroscopic presence of a meshoma, and patients’ description of his/her clinical situation (normal, mild, moderate, severely or intense pain). Selection of parameters was based on prognostic factors for developing postoperative pain described in available published literature [2, 9, 12, 14, 16–18, 23, 24].

POP patients were asked to complete multiple questionnaires at baseline including the Patient Global Impression of Change (PGI-C), which contains a 7-point Likert scale from *no change or worsening* to *very much better, a substantial change.* Patients select their answer concerning the change on activity, symptoms, emotions and general quality of life, related to the patient’s mesh-related symptoms [25]. The answers of *better and a worthwhile change* and *very much better and a substantial change* were defined as improved.

### Primary outcome measure

For both CPIP patients as for POP patients postoperative pain was used as primary outcome measure. Outcomes were categorized as improved, unchanged or worsened compared to the preoperative symptoms, as described by patients. Mesh revision was considered successful when symptoms were described as improved. An unchanged or worsened outcome was considered unsuccessful.

### Statistical methods

Data were analyzed and graphs were created using SPSS version 26.0 software (SPSS Inc., Chicago, IL, US). Patient characteristics and intraoperative variables were tested for confounding properties by univariate analysis. Significant confounders (*p* < 0.05) were included as covariates in a multivariable logistic regression model as a means to determine the association between potential prognostic variables and outcome measures. The corresponding odds ratios (OR) and 95% confidence intervals (95% CI) were calculated. Significant confounders (*p* < 0.05) were combined when necessary in a multivariable logistic regression model using a backward stepwise regression method. Risk/benefit profile was assessed using *Chi-square* test. Continuous data were expressed as mean, minimum, maximum and standard deviation (SD). Dichotomous or categorical data were expressed as frequencies and percentage points. Characteristics with more than 20% missing data were omitted from analysis. Statistical significance (alpha) was set at *p* = 0.05.

## Results

### Demographics

A total of 221 patients for CPIP and 59 patients for vaginal complaints after mesh implant were included in the dataset.

#### Inguinal mesh revision

Data of the patients included in the univariate analyses are presented in Table 1. Majority of patients were male (86%) and aged 45 to 65 years. Median time between implantation and explantation of the PP mesh was 3.9 years, median follow-up time was 0.1 years, corresponding the standard 4–6 weeks follow-up at the outpatient clinic post-surgery. 41% of revised meshes were revised in full. In 7% a macroscopically identified meshoma was present, defined as folding and wrinkling of the inserted mesh, varying from a mass-like density to a more subtle wrinkling or fibrosis [26].

**Table 1:** Patient demographics Inguinal mesh revision (*N* = 221)

	N (%)
Female	31 (14)
Male	190 (86)
Smoker	69 (31)
Non-smoker	84 (38)
Ex-smoker	67 (30)
Unknown	1 (0)
Age < 45 years	41 (19)
Age 45–65 years	148 (67)
Age > 65 years	32 (14)
Mesh removal < 50%	12 (6)
Mesh removal 50–99%	46 (21)
Mesh removal 100%	91 (41)
Percentage mesh removal unknown	72 (33)

	Minimum	Maximum	Mean	Std. Deviation
BMI (kg/m^2^)	17.0	45.4	25.9	4.0

	Minimum	Maximum	Median
Time between mesh placement and revision (years)	0.2	31.3	3.9
Time between mesh revision surgery and follow-up (years)	0.0	1.6	0.1
	Yes (%)	No (%)	
Meshoma, macroscopic presence	16 (7)	205 (93)	
NSAIDs use	63 (29)	157 (71)	
Opioid use	58 (26)	162 (73)	

### Vaginal mesh revision

Data of the patients who underwent vaginal mesh revision and were included in the univariate analyses, are presented in Table 2. Age of this patient group ranged from 37 to 77 years old. Patients had a mean BMI of 26.4 kg/m2 and 58% was overweight (34 with a BMI ≥ 25 of which 7 are obese with a BMI ≥ 30 kg/m2). Median time between mesh placement and revision surgery was 4.2 years. Approximately half of patients was sexually active (47%), had at least one comorbidity (48%) and reported symptoms as *better* or *very much better* (47%). Only 5% smoked during time of inclusion.

**Table 2:** Patient demographics Vaginal mesh revision (*N* = 59)

	Minimum	Maximum	Mean	Std. Deviation
BMI (kg/m^2^)	19.4	39.8	26.4	4.2
Abdominal surgeries in the past (n)	0	4	1.10	1.1
Surgeries for (partial) mesh removal (n)	1	17	2	2.4

	Minimum	Maximum	Median	
Age (years)	38	83	63	
Time between mesh placement and revision (years)	0.2	25.8	4.2	
Time between mesh revision surgery and follow-up (years)	0.2	6.3	1.7	

	Yes (%)	No (%)	Unknown (%)	
Smoking	3 (5)	56 (95)		
Vaginal delivery	57 (96)	1 (2)	1 (2)	
Sexually active	27 (46)	22 (37)	10 (17)	
Comorbidities	28 (48)	29 (49)	2 (3)	
PGI-C (reported *better* or *very much better*)	28 (47)	27 (46)	4 (7)	

### Primary outcome results

After revision of mesh because of CPIP, 152 of 221 patients reported improvement of symptoms (69%), 34 reported unchanged symptoms (15%) and 5 reported worsening of symptoms (2%). Unfortunately, 30 of 221 patients (14%) did not report the postoperative status of their symptoms at follow-up. Whether mesh was partially or completely removed did not have a statistically significant effect on reported improvement of symptoms (both 80% positive improvement, but 41% of cases either missing reported improvement or unknown percentage of removal). Results of partial versus complete removal are shown in Table 3.

**Table 3:** Improvement of pain after partial versus complete removal of inguinal mesh (n, (%))

	Improved	Unchanged	Worsened	Total
Partial removal	40 (80)	9 (18)	1 (2)	50 (100)
Complete removal	64 (80)	13 (16)	3 (4)	80 (100)
Total	104 (80)	22 (17)	4 (3)	130 (100)

Vaginal mesh revision resulted in 44 of 59 patients describing improvement of symptoms (75%), 7 patients describing unchanged symptoms (12%) and 3 describing worsening of symptoms (5%). 5 of 59 patients (8%) did not report the postoperative status of their symptoms at follow-up.

### Prediction analysis

With intent of a multivariable analysis, predefined predictors were analyzed for univariable association with improvement of pain after revision surgery. The OR and 95% CI of both inguinal mesh and vaginal mesh predictors with < 20% missing values are presented in Table 4.

**Table 4:** Univariable analysis—prediction of improvement of pain

Predictor	Inguinal mesh		Vaginal mesh	
	OR	*p*-value	OR	*P*-value
Gender (male vs. female)	0.83	0.718		
Age (years)	1.00	0.917	1.02	0.572
BMI (kg/m^2^)	1.02	0.599	1.01	0.884
Smoking (yes vs. no/ex-smoker)	1.22	0.385		
Meshoma, macroscopic presence (yes vs. no)	0.69	0.643		
Sexually active (yes vs. no)			1.17	0.845
Comorbidity (yes vs. no)			0.48	0.337
Revision surgery (N)			0.97	0.817
Prior abdominal surgery (N)			0.64	0.154
Visual Analogue Scale (per point)			0.75	0.331
Time between mesh placement and revision (per year)	1.17	0.008*	1.02	0.815
Time between mesh revision and follow-up (per year)	0.43	0.379	0.76	0.257

*statistically significant predictor with *p*-value < 0.05

### Multivariable analysis

#### Inguinal mesh revision

When all predefined and collected predictors were included, no participants proved suitable for logistic regression analysis in this small sample size, due to a considerable number of missing values. Therefore, multivariable analysis was only performed with a priori selected predictors for which more than 80% of data was available. The OR and 95% CI of predictors suitable for analysis are presented in Table 5.

**Table 5:** Multivariable analysis inguinal mesh revision—correlation with symptom improvement

Predictor	Odds ratio (OR)	95% CI
Meshoma, macroscopic presence (yes vs. no)	2.30	0.27 – 19.44
Time between mesh placement and revision (per year)	1.19*	1.05 – 1.34*
Smoking (smoker vs. ex-smoker)	0.79	0.30 – 2.06
Smoking (non-smoker vs. ex-smoker)	0.92	0.35 – 2.40

*p < 0.05

**Fig. 1:**
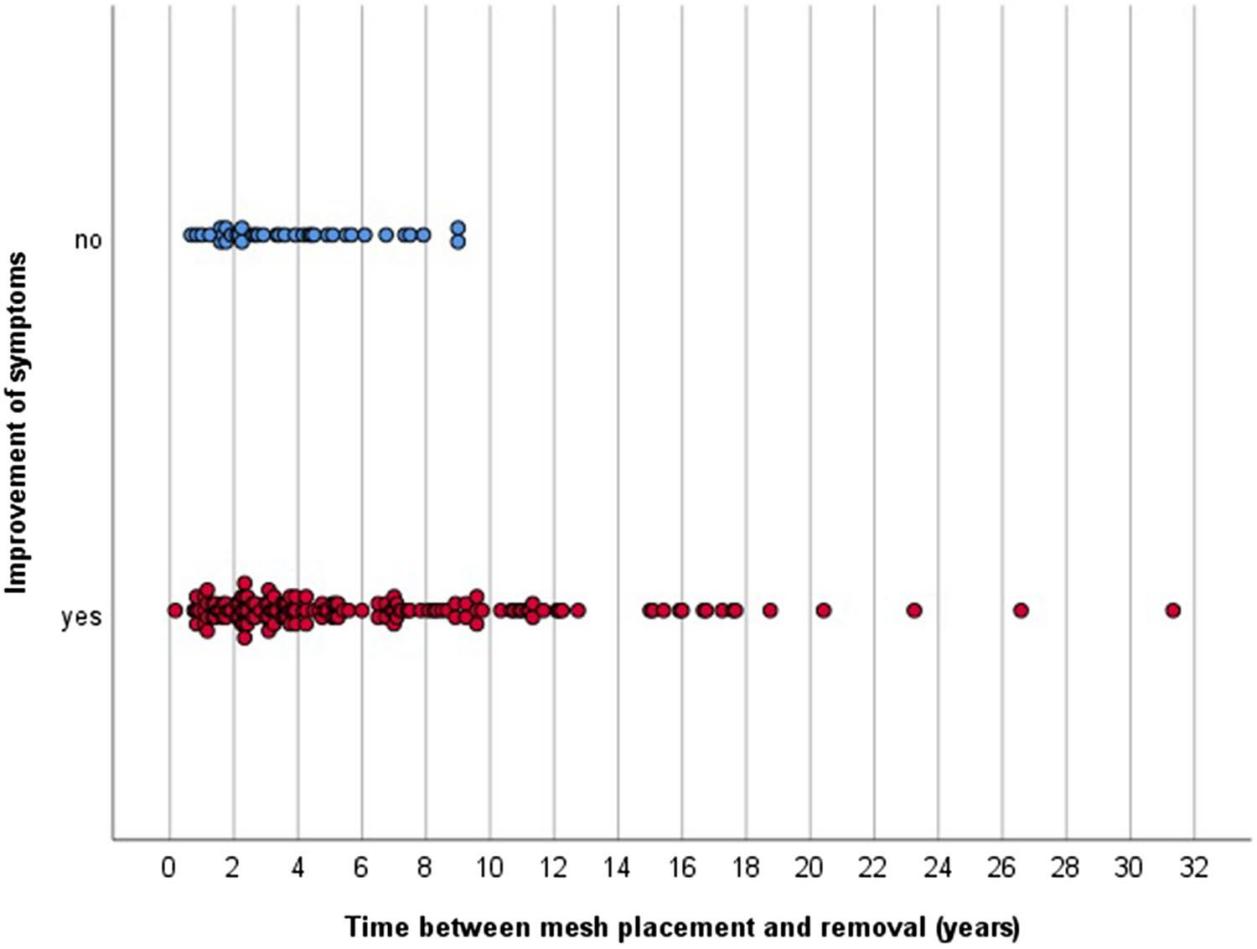
Improvement of symptoms displayed by time between mesh placement and removal

Time between mesh placement and revision surgery was the only statistically significant variable, meaning that in patients who suffered from pain symptoms for a longer period, mesh removal was more likely to lead to an improvement of CPIP. Figure 1 presents a scatter plot that sets out improvement of mesh revision versus the number of years after implantation of the PP mesh. In the present series, all patients who underwent revision surgery after a period of at least 9 years since mesh placement, remarkably had improvement of symptoms, which explains the results of the multivariable analysis.

#### Vaginal mesh revision

None of the predictors were univariable statistically significant. Due to a small sample size and a considerable number of missing values, multivariable analysis of the initially chosen predictors was not possible. Also, sub-analysis of different surgical techniques and partial versus complete removal of mesh was not possible. We, therefore, chose to estimate associations for the four continuous predictors. The OR and 95%CI are presented in Table 6. A sub-analysis of different surgical techniques and partial or complete removal of mesh was not possible, due to missing values.

**Table 6:** Multivariable analysis vaginal mesh revision—correlation with pain improvement

Predictor	Odds ratio (OR)	95% CI
Age (per year)	1.14	0.95–1.39
Time between mesh placement and revision (per year)	1.31	0.72–2.39
Time between mesh removal surgery and follow-up (per year)	1.50	0.20–11.08
Visual Analogue Scale (per point)	0.88	0.39–1.96

### Risk–benefit profile

**Fig. 2:**
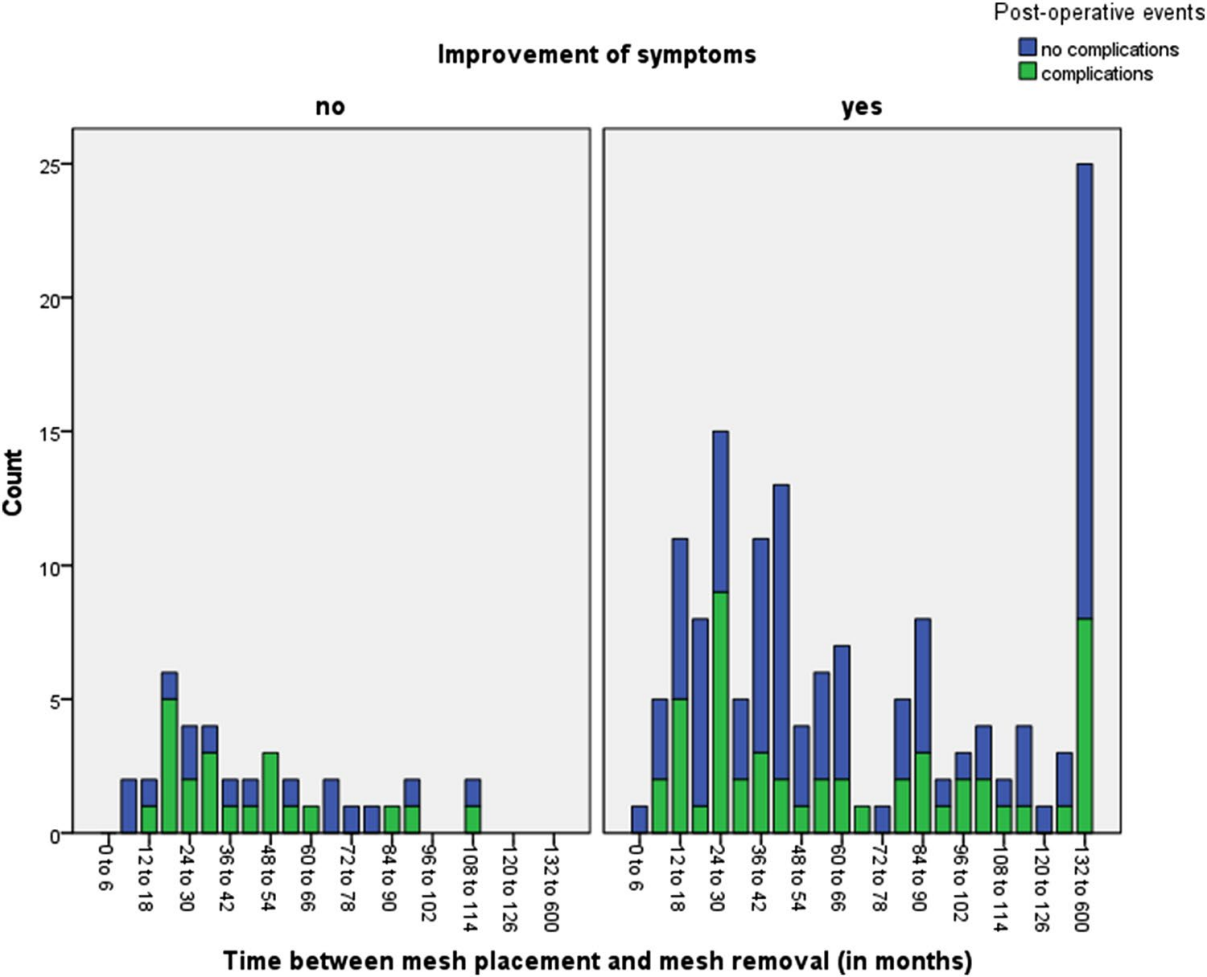
Risk–benefit profile, distribution of complications over time within participants with and without improvement of symptoms after revision surgery

After a predictive value of time between mesh placement and revision surgery for better clinical outcome was seen, the assumption arose that a threshold time-point after mesh placement for higher chances on improvement of symptoms than chances on complications could be found. The total complication rate in all participants was 37.6%. It should be noted that these complications range from small hematoma and mild wound pain to, for example, recurrent hernia inguinalis. A higher rate of complications was seen in the small group of participants without improvement of symptoms (57%), compared to 35% in patients with improvement of symptoms. Figure 2 shows the distribution of these complications per 6 months of time between mesh placement and mesh removal. No clear time-point could be identified where benefits outweigh risks of the surgery, since in this cohort the levels of complications were lower than the levels of improvement of symptoms in almost all time-cohorts and no specific trend in these complication rates was seen. Figure 2 shows an apparent turning point at 70 months between placement and revision of mesh. An OR of 2.86 (95% CI 1.18–6.94, *p* = 0.017) was found for improvement of pain in patients operated after 70 months, compared to patients operated before 70 months of time between mesh placement and revision.

## Discussion

### Main findings

In the present retrospective case series predictors for the improvement of pain after (partial) removal of a PP implant from the inguinal or vaginal region are identified. The multivariable analysis for inguinal hernia patients concluded that only the time between insertion of an inguinal implant and the removal of that implant has a positive predictive value for improvement of pain after revision surgery. This signifies that inguinal hernia patients with later onset of pain or patients that have endured pain for a longer period of time, have a higher chance of a positive effect of the mesh revision with an OR of 1.19 per year. A turning point in chances of risks and benefits for revision surgery was demonstrated at 70 months after mesh implantation, with improved outcomes for patients with revision surgery ≥ 70 months (OR 2.86). A possible explanation for this effect is that patients indicated for revision surgery after more years, have a better indication in comparison to patients who are indicated earlier, as in the latter other factors contributing to the pain may play a role. In this case, pain is a comorbidity, which results in the assumption that it is mesh related, whereas it is not. Another possibility is that the body is capable of physical adjustment to pain regardless of the cause, localization, type of surgery et cetera (e.g., by forming a meshoma or neuroma) but in some cases in time these adjustments are of insufficient effect and pain becomes present. In addition, it is important to realize that meshes induce a foreign body response (FBR), which can result in fibrosis and pain at several years after implantation. For the limited group of POP repair patients with missing values, no statistically significant association between assumed predicting variables and improvement of pain are found.

### Interpretation

(Partial) removal of a PP implant does not always result in complete relief of symptoms, as is documented by the number of 11.5 to 27% unresolved pain symptoms [11, 27, 28] and 36% of patients with poor to moderate outcome following inguinal mesh revision[9]. Successful outcome of mesh revision is multifactorial and thus difficult to predict [8, 17].

Previous studies demonstrated several variables predicting improvement of symptoms after removal of PP mesh implants. Chances of relief of CPIP after mesh repair by mesh removal may be increased when a meshoma is (identified and) removed and the operation is carried out with spinal anesthesia. In contrast, preoperative use of opioids may limit success rates of mesh revision with or without neurectomy, as demonstrated by a single retrospective study [29]. In addition, Danford et al. described that patients with a history of chronic pelvic pain, are more likely to not respond to revision surgery (OR 0.28) [27]. We think that explains why the outcome improves if this surgery is performed later after inguinal mesh placement surgery, as on the short term, patients with chronic pain may be overrepresented in those indicated for removal surgery.

Apart from the reported factors above, an association was expected between higher pain scores [17], exposure and/or erosion of the implant, the presence of vaginal and/or pelvic pain, dyspareunia [8, 18, 30], and a foreign body feeling [9]. A foreign body feeling can be described as a mechanical pressure and sensation of tightness, for example in the groin or abdominal area. In theory, this feeling can be resolved by removal of the foreign body by eliminating the cause of mechanical pressure.

Another explanation for improvement of pain after later explantation may be an ongoing Foreign Body Reaction (FBR). Although a PP implant does not seem to induce a systemic autoimmune response [31, 32], and FBR is not the only known factor for occurrence of complications, the local FBR is likely to add to the fibrotic tissue and scarring formation that can result in pain [33]. This local response can persist up to more than 10 years after implantation [7, 31, 33]. A local ongoing inflammatory response was previously demonstrated by histopathologic findings of explanted meshes [33] and in current literature, it is unclear whether this FBR ever resolves [31], but we assume the FBR to stay active as long as the mesh stays in the body. Long-term symptoms including CPIP, vaginal and/or pelvic pain and dyspareunia are thereby not completely unexpected. Since an association of ongoing inflammatory response to PP implants and local pain may be present, removal of the mesh may hypothetically lead to an improvement of complaints [18]. We hypothesize that the identified association of a higher chance on pain improvement and a longer period of time between implantation and explantation of the mesh in the present series, could be (partially) explained by this ongoing FBR. We propose that patients with CPIP and a suspicion of the present pain being related to the inserted mesh, should always be considered for revision surgery, independent from the time between mesh placement and presentation of the patient.

Nowadays, caregivers and patients decide together whether or not revision of a PP implant is indicated, and the surgeon assesses the possibility of improvement of pain in a specific patient. The patient deliberates whether present pain is severe and invalidating to such a degree that the risk of surgery outweighs the potentially limited effect on pain and other bothersome symptoms. In addition, the caregiver performs physical examination including palpation and/or vaginal examination to assess if the pain is related to the location, position of the implant, or tension or excessive scar tissue surrounding the implant and assesses if an exposure is present in case of a vaginal mesh. The present study indicates that a longer time period of mesh implantation and presentation at the outpatient clinic does not contra-indicate mesh revision. Apart from associations with physical examination and the knowledge of possible risks and/or consequences of (partial) removal surgery, the experience of the surgeon or gynecologist, history of the patient including duration and severity of pain, sexual dysfunction [30], and comorbidities also play an important role in the decision-making process. In patients that have had POP surgery, another factor taken into consideration is the chance of recurrent symptomatic POP (and/or SUI) after revision surgery. Available literature reports recurrence rates of POP from 8 to 31% depending on the operated vaginal compartment and partial or complete removal [11, 13], and recurrent symptoms of urinary incontinence, dyspareunia and pelvic pain (24, 16 and 12% respectively) [8]. The possibility of recurrence should be discussed with a patient before revision surgery is carried out, but should not restrain the caregiver from the indication of surgery when an association between pain and mesh is expected.

To assist surgeons and patients in this decision-making process, we attempted to make a risk–benefit profile to identify a point in time in our inguinal hernia mesh surgery cohort where improvement rates of revision surgery become higher than expected complication rates. To our knowledge, such a risk–benefit analysis has not been carried out before. However, we did not find a trend in complication rates, nor a cutoff point for better clinical outcomes compared to complication rates. Therefore, we cannot advise on specific timing of revision surgery based on our present data.

### Strengths and limitations

To our knowledge, this study is exclusive in its design, because we identify predictors for mesh revision surgery for two different indications. As both participating centers are experts in revision surgery, the practice variation and learning curve that are specific for this type of surgery, will have had minimal impact on our findings. Furthermore, to our knowledge the formation of a risk/benefit profile for revision surgery of mesh has not been attempted before. Certain limitations of this study have to be addressed. Both cohort have small group sizes (especially the POP repair group) and due to the retrospective character of this study there are some missing data. This is inherently related to the study design and it is important to realize that a prospective study could generate more insightful data. However, patients presenting with mesh related complications cannot wait for such prospective study. Especially not POP patients since the number of patients will diminish over time as the U.S. Food and Drug Administration has stopped manufacturers to commercialize vaginal mesh procedures.

## Conclusion

In conclusion, it seems difficult to predict who will benefit from (partial) mesh removal surgery and who will not, as we could not identify a set of predictors for surgical success. Given the 69–75% rate of success, it seems that surgeons in expert centers are successful in indicating patients for revision surgery, based on factors that cannot be captured in quantifiable parameters. This is a plea to perform revision surgery in a center of excellence.

The one factor that was identified for a positive prediction on improvement of pain after removal of an inguinal PP implant, was the duration of time between placement of mesh and revision surgery with an OR of 1.19 per year. A turning point for higher chances on improvement of pain with revision surgery was found at 70 months after initial placement of the mesh using a risk/benefit profile. No other variables were proven significant for prediction of improvement of pain.

Patient-specific consultation and informed consent is necessary to determine whether revision surgery is preferred over conservative treatments. Follow-up of patients with CPIP or chronic pain after vaginal PP implant should be performed by an expert in this field, preferably in an expertise center where multidisciplinary outpatient clinic carousels are present[34].

When counseling patients for mesh revision surgery in case of bothersome symptoms, caregivers should explain the non-negligible complication risks and recurrence rates, but should not avoid revision surgery based on these factors, since (partial) removal of mesh has proven to be successful in pain and symptom relief[9, 11, 13, 27, 28]. Above this, current case series shows that a longer duration between implantation of inguinal mesh and revision surgery of at least 70 months has a higher chance on improvement of pain, meaning caregivers should not refrain from indicating surgery based on a longer duration of symptoms when an association between symptoms and the location of the mesh is expected.

## Data Availability

The data that support the findings of this study are available from the corresponding author, VR, upon reasonable request.
